# Multi-Omics Analysis Revealed the Molecular Mechanisms Affecting Average Daily Gain in Cattle

**DOI:** 10.3390/ijms26052343

**Published:** 2025-03-06

**Authors:** Mingjuan Gu, Hongyu Jiang, Fengying Ma, Shuai Li, Yaqiang Guo, Lin Zhu, Caixia Shi, Risu Na, Yu Wang, Wenguang Zhang

**Affiliations:** 1 College of Animal Science, Inner Mongolia Agricultural University, Hohhot 010010, China; gmj0119@yeah.net (M.G.); 15894949495@163.com (H.J.); fengyingma1997@163.com (F.M.); lishuai@emails.imau.edu.cn (S.L.); gggyaqiang@163.com (Y.G.); zhulinynacxhs@163.com (L.Z.); shicx98@163.com (C.S.); narisu@swu.edu.cn (R.N.); 2 College of Veterinary Medicine, Inner Mongolia Agricultural University, Hohhot 010010, China; 3 College of Life Science, Inner Mongolia Agricultural University, Hohhot 010010, China

**Keywords:** average daily gain, Angus cattle, 16S rRNA, metabolome, transcriptome

## Abstract

The average daily gain (ADG) is a critical index for evaluating growth rates in cattle and is closely linked to the economic benefits of the cattle industry. Heredity is one of the factors affecting the daily gain of cattle. However, the molecular mechanisms regulating ADG remain incompletely understood. This study aimed to systematically unravel the molecular mechanisms underlying the divergence in ADG between high average daily gain (HADG) and low average daily gain (LADG) Angus cattle through integrated multi-omics analyses (microbiome, metabolome, and transcriptome), hypothesizing that the gut microbiota–host gene–metabolism axis is a key regulatory network driving ADG divergence. Thirty Angus cattle were classified according to their HADG and LADG. Fecal and serum samples were collected for 16S, fecal metabolome, and blood transcriptome analysis. The results showed that compared with the LADG group, the abundance of Firmicutes increased in the HADG group, while the abundance of Bacteroidetes and Proteobacteria decreased. Metabolomics and transcriptomic analysis revealed that KEGG pathways associated with differentially expressed genes (DEGs) and differentially abundant metabolites (DAMs) were enriched in bile acid metabolism. Spearman correlation analysis showed that *Oscillospira* was positively correlated with *ZBTB20* and negatively correlated with *RADIL*. *ZBTB20* was negatively correlated with *dgA-11_gut_group*. This study analyzed the regulatory mechanism of average daily gain of beef cattle from genetic, metabolic, and microbial levels, providing a theoretical basis for analyzing the mechanism of differential daily gain of beef cattle, and has important significance for improving the production performance of beef cattle. The multi-omics network provides biomarker foundations for machine learning-based ADG prediction models, offering potential applications in precision breeding. While these biomarkers show promise for precision breeding, their causal roles require further validation. The conclusions are derived from a single breed (Angus) and gender (castrated males). Future studies should include females and diverse breeds to assess generalizability.

## 1. Introduction

Beef, renowned for its rich nutritional value, is popular among consumers worldwide, with its consumption rising, particularly in developing nations [1]. The average daily gain (ADG), as a key trait of beef cattle breeding, is closely related to economic benefits. An increase in ADG is helpful to improve the production efficiency and economic benefit of the beef cattle industry. Achieving the target market weight in a shorter time is possible for cattle with higher ADG compared to those with lower ADG, thus significantly cutting down on feeding costs. Many factors can affect cattle ADG, including nutrition, genetics, environment, management, health, and other factors [2]. For example, the daily gain of calves can be significantly improved by feeding the calves with astragalus root extract [3]. In addition, cattle temperament affects ADG, with cattle that are quieter and calmer during handling having greater average daily gains than cattle that become agitated during routine handling [4]. Daily gain is closely related to the digestion and absorption capacity of the small intestine. Studies have shown that jejunum gene expression profiles are significantly different in cattle of high and low daily gain [5]. Moreover, Sunirmal Sheet et al. conducted transcriptome analysis on three distinct skeletal muscles of Hanwoo cattle, including the longissimus dorsi (LD), semimembranosus (SB), and psoas major (PM), to explore genetic influences on ADG variation [6]. However, the molecular mechanisms regulating ADG remain incompletely understood, partly due to limitations in sample size, technical approaches, and data integration.

With the advancement of high-throughput sequencing and omics technologies (e.g., RNA sequencing, microbiome analysis, and metabolomics), these approaches have become common methodologies in growth and development of livestock research. Gut microbiota plays an important role in the average daily gain of livestock [7]. The intestinal microbiome influences gut and systemic health through its metabolites [8]. The fecal metabolome is a “functional reading” that links microbial activity to phenotypes [9]. The blood transcriptome reflects systemic host response [10]. Most published studies have focused on single-omics approaches. While individual omics analyses have significantly contributed to the mechanisms regulating growth and development in domestic animals, they are somewhat limited because they focus on a single domain. The use of multi-omics measurements can yield more information than single omics, resulting in insightful conclusions that cannot be derived from any single omics technique [11]. For this reason, multi-omics approaches are gaining popularity. In previous studies exploring the mechanism of ADG in ruminants, key gaps still exist, such as small sample size (*n* < 20) limiting the universality, single-omics perspective failing to reveal cross-layer regulatory networks, and insufficient integration of multi-omics data hindering the verification of critical pathways [7,12]. The physiological mechanism of regulating the average daily gain of beef cattle is still a complex scientific problem, and no single omics can fully uncover the mechanism. Integrating multiple omics strategies is beneficial to further understand the regulatory mechanism of differences in average daily gain of beef cattle from a systematic and holistic perspective. This study combines an expanded cohort (n = 30) with integrative microbiome–metabolome–transcriptome analyses and Spearman/Mantel tests to systematically decode ADG divergence, bridging technical and integrative gaps in current research. Therefore, this study aimed to answer how gut microbiota composition, host gene expression, and metabolite profiles differ between high- and low-ADG cattle. How do these differences synergistically regulate ADG through cross-omics interactions?

In this study, the microbiome, metabolome, and transcriptome of Angus cattle with high and low daily gain were analyzed under the same feeding management and dietary conditions, and the molecular mechanism of the difference in daily gain was investigated. Fecal and serum samples from 30 Angus steers (15 high-/low-ADG each) were subjected to 16S rRNA sequencing (microbiome), untargeted metabolomics (UHPLC-MS), and whole-blood transcriptomics (RNA-seq). Cross-omics regulatory networks were constructed via Spearman correlation analysis. This study provides a theoretical basis for analyzing the mechanism of differential daily gain of beef cattle and has important significance for improving the production performance of beef cattle.

## 2. Results

### 2.1. Comparison of the Fecal Microbiotas of High- and Low-Daily-Gain Performance Groups

**Figure 1:**
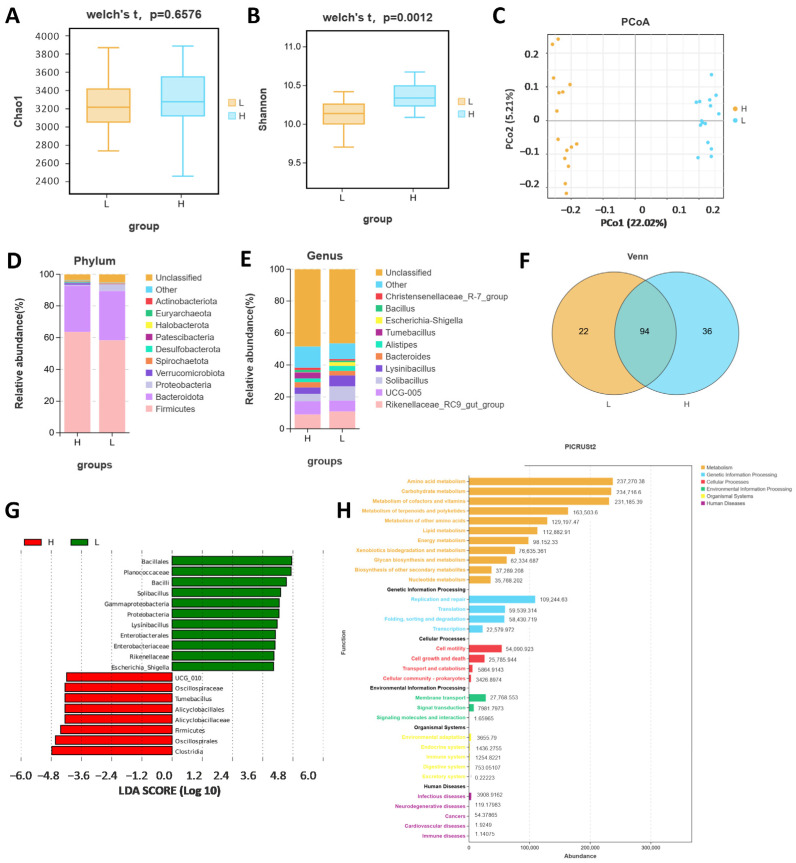
The 16S sequencing of fecal samples from HADG and LADG cattle. The α-diversity (alpha diversity) plots with chao1 index (**A**) and Shannon index (**B**). (**C**) Principal coordinate analysis (PCoA) of fecal microbial communities based on the Bray–Curtis distance. (**D**) The distribution of microbiota at the phylum level. (**E**) The distribution of microbiota at the genus level. (**F**) Venn diagram. (**G**) Histogram of LDA value distribution of LEfSe analysis. (**H**) PICRUSt2 analysis.

A total of 30 Angus cattle were tested, with 15 randomly selected from high- and low-daily-gain groups, respectively. To assess whether the amount of sequencing in this study was adequate to capture the diversity of the original microbial communities, the 16S rRNA sequencing data were analyzed for α-diversity. The Chao 1 index, which measures species richness, indicated no significant differences between the high- and low-daily-gain groups (*p* = 0.6576, Figure 1A). Conversely, the Shannon index, which also reflects species richness, revealed significant differences between the two groups (*p* = 0.0012, Figure 1B). Statistical analysis of α-diversity indices revealed that the high-daily-gain group had greater community and species richness compared to the low-daily-gain group (Figure 1A,B). Although there were similarities in microbial community diversity among the groups, differences in community richness were observed. Beta diversity, which involves comparing biodiversity across different samples, was assessed using principal coordinate analysis (PCoA) (Figure 1C). The distribution of fecal microbiota composition between the high-daily-gain group and the low-daily-gain group is distinctly separated, showing significant differences in bacterial communities between the two groups. Species composition analysis provides insight into the community structure of samples at various taxonomic levels. Figure 1D,E illustrate the results of species annotation at the phylum and genus levels for the two groups, as shown by 16S rRNA sequencing. At the phylum level, Firmicutes was the dominant member of the gut microbiota in all individuals, constituting between 52.6% and 77.6% of all phyla, followed by Bacteroidota and Proteobacteria. Compared with the low-daily-increase group, the abundance of Firmicutes in the high-daily-increase group increased, while the abundance of Bacteroidota and Proteobacteria decreased. We selected the top 10 genera at the genus level. The category Others contains the remaining species, while Unassigned is for those without classification or annotation. At the genus level, the dominant bacteria in the gut of high daily gain for cattle included 10 species, such as *Rikenellaceae_RC9_gut_group*, *UCG-005*, *Solibacillus*, *Lysinibacillus*, *Bacteroides*, and *Alistipes* (Figure 1E). At the genus level, a total of 152 genera were identified, of which 94 were shared between the two groups (Figure 1F). Biomarkers with significant differences between groups, from the phylum to genus level, were identified using LefSe analysis (LDA score > 4) (Figure 1G). A total of 19 different microflora strains were isolated from the intestinal contents, according to the results, including 2 phyla, 3 class, 4 orders, 6 families, and 4 genera. Clostridia was the most abundant marker in intestinal contents in the HADG. Bacillales was the most abundant market in the LADG. The predicted functions of gut bacteria were analyzed using PICRUSt2. A total of 32 of the most important functions were detected for gut bacteria (Figure 1H). The top 10 functions were amino acid metabolism, carbohydrate metabolism, metabolism of cofactors and vitamins, metabolism of terpenoids and polyketides, metabolism of other amino acids, lipid metabolism, energy metabolism, xenobiotics biodegradation and metabolism, glycan biosynthesis and metabolism, and biosynthesis of other secondary metabolites.

### 2.2. Comparison of the Fecal Metabolites of High- and Low-Daily-Gain Performance Groups

**Figure 2:**
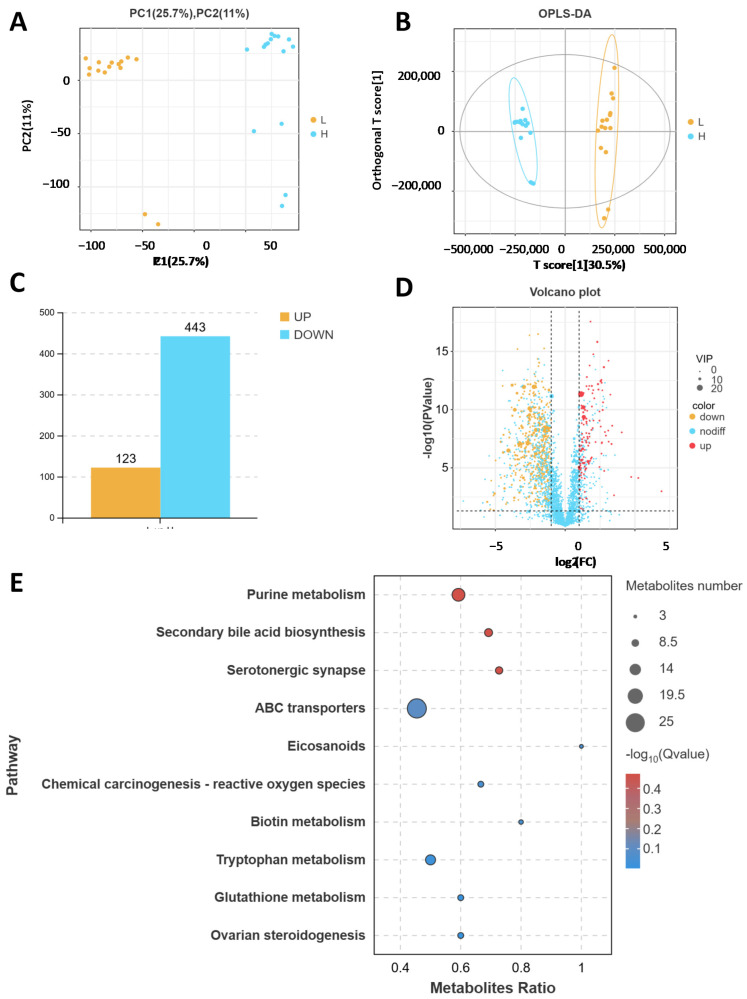
Metabolome analysis of fecal samples from HADG and LADG cattle. (**A**) PCA score plot of the metabolome. (**B**) OPLS-DA score plot of all the metabolite features. (**C**) Number of up- and down-regulated differentially abundant metabolites. (**D**) Volcano diagram of differentially abundant metabolites. (**E**) KEGG enrichment analysis of differentially abundant metabolites from HADG and LADG cattle.

A non-targeted metabolomics analysis was performed on 30 cattle fecal samples. To compare the metabolomic analysis results of the two groups, Figure 2A,B shows the results of principal component analysis (PCA) and orthogonal partial least squares discriminant analysis (OPLS-DA) analyses. The two models showed significant clustering, indicating there was obvious separation between the two groups. In total, 566 differentially expressed metabolites were identified, with 123 metabolites upregulated and 443 downregulated in the HADG group compared with the levels in the LADG group ((VIP) ≥ 1, *p* < 0.05 and FC ≥ 2) (Figure 2C,D). In addition, we found through KEGG enrichment analysis that these metabolites were involved in purine metabolism, secondary bile acid biosynthesis, serotonergic synapse, and ABC transporters (Figure 2E).

### 2.3. Comparison of the Blood Transcriptome of High- and Low-Daily-Gain Performance Groups

**Figure 3:**
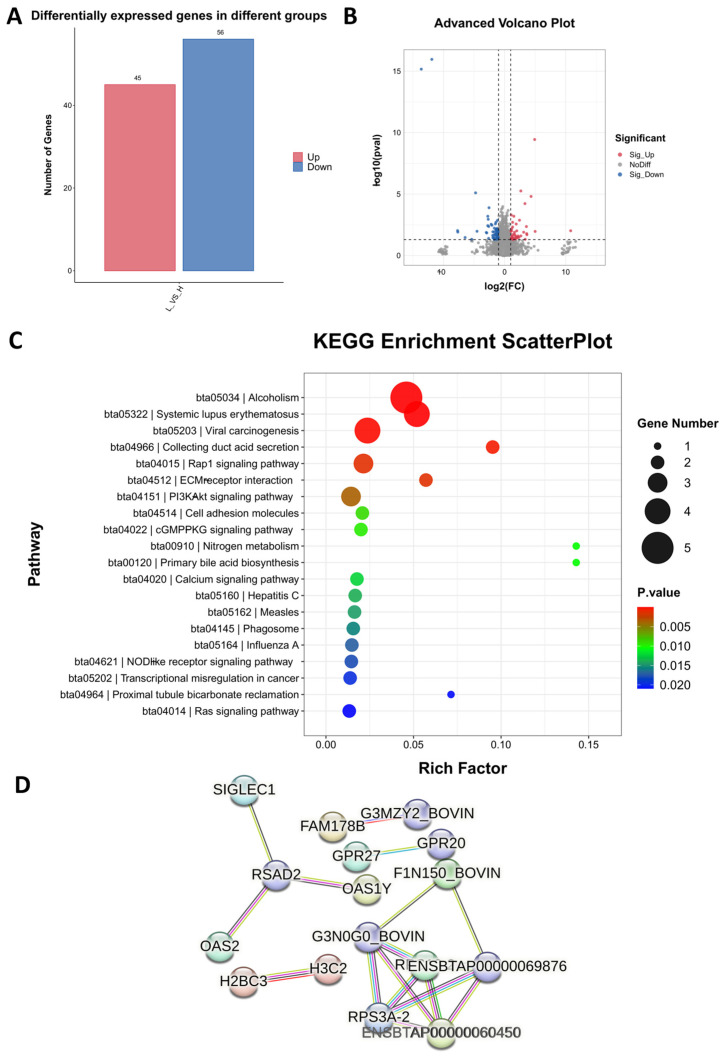
Transcriptome analysis of blood samples from HADG and LADG cattle. (**A**) The number of up- and down-regulated differentially expressed genes (DEGs). (**B**) Volcano diagram of the differentially expressed genes. (**C**) Differentially expressed gene KEGG enrichment. (**D**) PPI network of differentially expressed genes.

To explore the molecular mechanism of the ADG of cattle, we used transcriptome sequencing technology to analyze the enrichment of DEGs and pathways in high and low daily weight gain in bovine blood. The results showed that there were 101 DEGs (FDR < 0.01; fold change > 2 or <−2), of which 45 were up-regulated and 56 were down-regulated (Figure 3A,B). Enrichment analysis showed that the top 10 signaling pathways were alcoholism, systemic lupus erythematosus, viral carcinogenesis, collecting duct acid secretion, rap1 signaling pathway, ECM–receptor interaction, PI3K-Akt signaling pathway, cell adhesion molecules, cGMP-PKG signaling pathway, and primary bile acid biosynthesis (Figure 3C). To anticipate how the identified DEGs interact, we utilized the online string platform to predict the protein–protein interaction network of differentially expressed genes (Figure 3D).

### 2.4. Integrative Crosstalk of Multi-Omics

**Figure 4:**
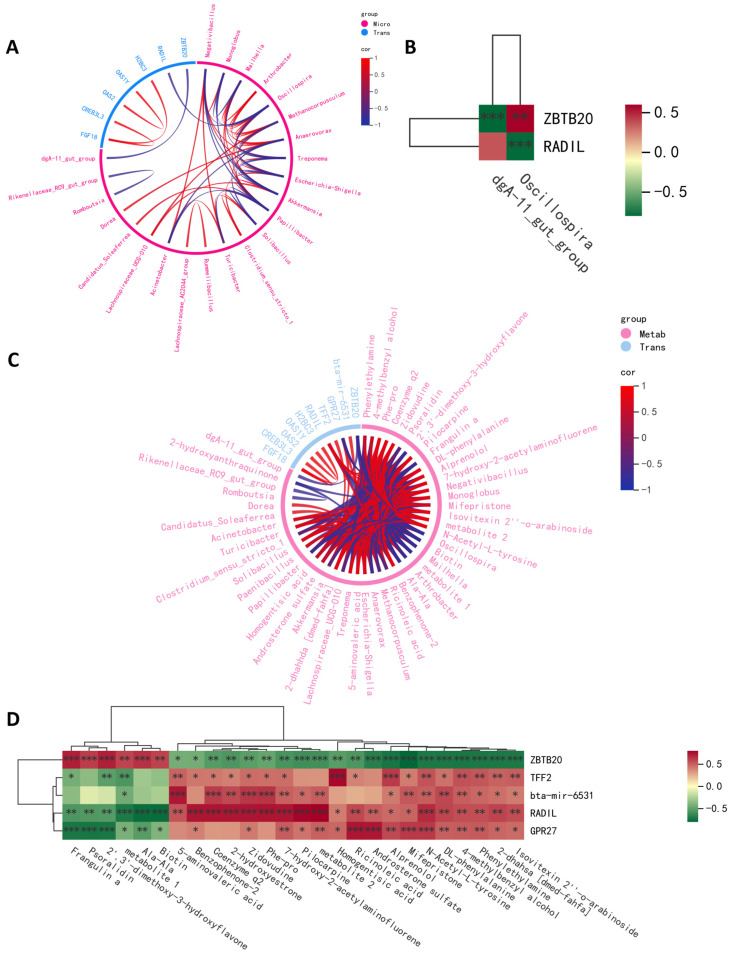
Multi-omics correlation analysis. (**A**) Spearman correlation chord plot of differential microbial and differentially expressed genes. (**B**) A heat map of the correlation analysis between differential microbial and differentially expressed genes. (**C**) Spearman correlation chord plot of differentially abundant metabolites and differentially expressed genes. (**D**) A heat map of the correlation analysis between differentially abundant metabolites and differentially expressed genes. metabolite 1 represents 6h-dibenzo[b,d]pyran, 3-(1,1-dimethylheptyl)-6a,7,10,10a-tetrahydro-1-methoxy-6,6,9-trimethyl-, (6ar,10ar)-; metabolite 2 represents 1h-pyrrole-3-propanoic acid, 5-[(1,2-dihydro-2-oxo-3h-indol-3-ylidene)methyl]-2,4-dimethyl-; * *p* < 0.05, ** *p* < 0.01, *** *p* < 0.001.

To study the link between gene and microbiota and metabolites in high- and low-daily-weight-gain cattle, we determined the significant correlations between DEGs and various differential microorganisms and metabolites. The relationship between DEGs and gut microbiota and metabolites was examined using Spearman’s correlation, which was visualized through a heatmap and a chord diagram (Figure 4). Significant gene–fecal microbiota and metabolite correlations were determined based on |r| > 0.69 and *p* < 0.05. The association analyses of differential gut microbiota and differential genes are presented in Figure 4A,B. We observed a positive correlation between *ZBTB20* and *Oscillospira* and a negative correlation between *RADIL* and *Oscillospira*. Additionally, we found a negative correlation between *ZBTB20* and *dgA-11_gut_group*. As for *RADIL*, there was no relationship between *RADIL* and *dgA-11_gut_group*. Association analyses of differentially expressed genes and differentially abundant metabolites demonstrated that *ZBTB20* and *RADIL* were associated with 26 fecal metabolites, and TFF2, bta-mir-6531, and GPR27 were associated with 20, 17, and 22 fecal metabolites.

**Figure 5:**
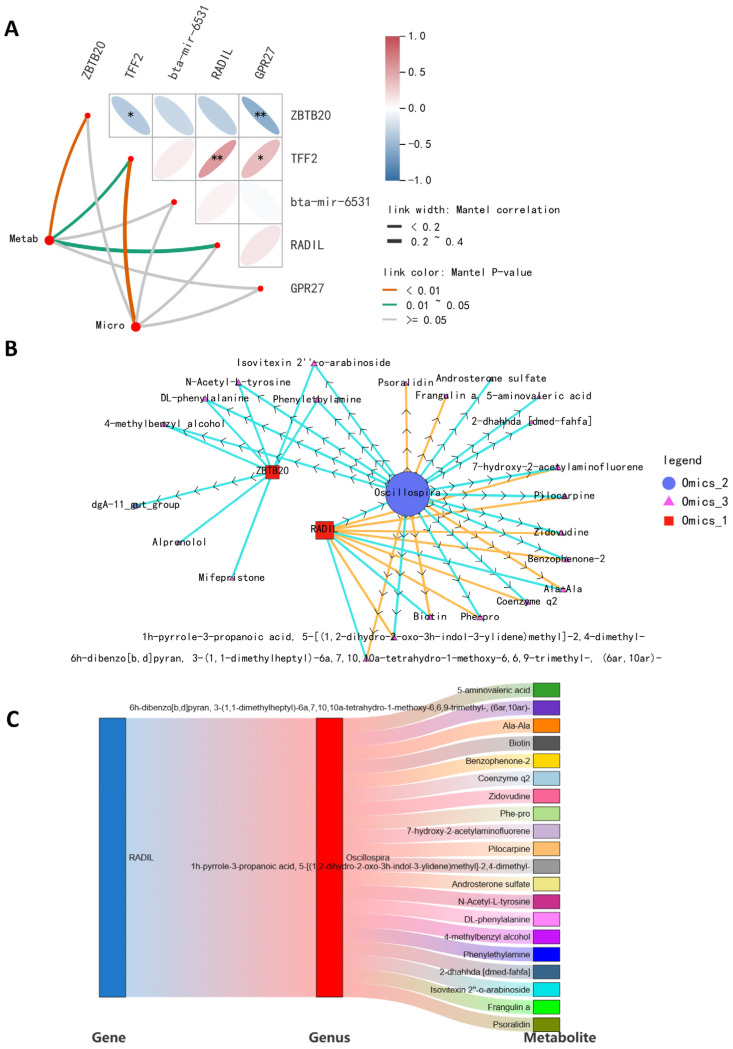
Integration of microbiome, transcriptome, and metabolome. (**A**) Mantel test correlation plot. The thickness of the line represents the size of the Mantel test correlation coefficient; the color of the line indicates the *p*-value of the Mantel test. * *p* < 0.05, ** *p* < 0.01. (**B**) A network diagram of microbiome, transcriptome, and metabolome. Different nodes in the diagram mark different microbiota, metabolites, or genes. The shape of the microbiota is circular, the shape of the metabolites is triangular, and the shape of the genes is square. (**C**) A Sankey diagram showing the correlation of genes–microorganisms–metabolites.

A comprehensive analysis of microbiome, metabolome, and transcriptome data can help elucidate the interactions between host gene expression, microbial composition, and the daily weight gain of cattle. It is generally believed that the high correlation may be caused by the relationship between different omics data, such as regulation and interaction, so the results based on the threshold sieve correlation can excavate the potential relationship. Therefore, we created a comprehensive multi-omics network using transcriptomics, metabolomics, and metagenomics data to demonstrate the functional connections between analytes within and across different omics datasets (Figure 5A). By constructing an interactive multi-omics correlation network, it was found that *RADIL* and *Oscillospira* played a central role in the daily weight gain of cattle (Figure 5B). The Sankey diagram further shows the linkages among the gut microbiome, fecal metabolites, and blood transcriptome (Figure 5C). These results suggest that *RADIL* may influence bovine daily weight gain by regulating metabolism through the regulation of intestinal enteric *Oscillospira* abundance.

## 3. Discussion

The economic advantages for the beef cattle industry are linked to average daily gain (ADG), making it a vital metric of production performance in commercial beef cattle, but the specific genetic mechanism of regulating ADG is not completely clear. Gaining a complete understanding of the genetic mechanisms related to key growth and livestock traits facilitates the discovery of new genes and genetic pathways that can be beneficial for selecting livestock for breeding. In recent years, multi-omics combined analysis technology has been gradually applied to the analysis of key factors of complex biological pathways, which can be verified and interpreted from different levels, and is more conducive to revealing the complex regulatory mechanisms of animal growth and development [13,14]. Hence, microbiome, metabolome, and transcriptome analyses were performed on high- and low-daily-gain Angus cattle in this study to provide novel molecular functions that may influence average daily gain in Angus cattle.

Gut microbiota significantly influences host health, metabolism, growth, and development [15,16]. The gut microbiome is crucial for maintaining cattle health and enhancing their performance [17,18]. Previous studies have shown that there are significant differences in the intestinal microbes of high- and low-daily-gain livestock. Zhou et al. indicated that goats with higher ADG harbored more abundant *Ruminococcus* in the rumen after compound enzyme preparation supplementation [19]. Yin et al. investigated the rumen and rectum microbial community in lambs with high and low ADG and revealed significant differences in gut microbes with different daily gain [7]. In pigs, ADG positively correlates with abundance of *Erysipelothrix*, *Streptomyces*, *Dubosiella*, *Parolsenella*, and *Adlercreutzia*, and negatively correlates with abundance of *Lactobacillus* and *Prevotella* [20]. Fang et al. found that the abundance of *Ruminococcaceae* and *Bacteroidales_S24-7_group* in the HADG was significantly higher than in the LADG, while *Eubacterium_coprostanoligenes_group*, *Christensenellaceae_R-7_group*, and *opportunistic pathogens* in the HADG were significantly lower than in the LADG in meat rabbits [21]. Huang et al. found that the *Prevotellaceae_NK3B31_group* and *Alistipes* were significantly associated with an increase in ADG in broilers [22]. In addition, *Lactobacillus* was positively correlated with ADG, while *Clostridium_sensu_stricto_1* was negatively correlated with ADG in post-hatching chicks [23]. This study found that *Clostridia* was the most abundant in intestinal contents in the HADG, and *Bacillales* was the most abundant in the LADG. *Clostridium* is strictly anaerobic breathing [24]. Interestingly, clostridium produces short-chain fatty acids as metabolic end products, such as propionic acid and butyric acid, which promote intestinal development and health in producing animals and are essential for improving their growth performance [25,26,27]. Studies have shown that butyrate supplementation can increase average daily gain in cattle [28]. This may be one of the reasons for the high daily weight gain of cattle.

Many studies have shown that changes in metabolite profiles are related to livestock performance [29,30,31]. This includes an analysis of the metabolome of livestock with differential daily gain. Feng and colleagues examined the metabolite profiles in sheep serum to identify the metabolic traits of sheep with varying average daily gains under identical management conditions [32]. Jiang et al. integrated analyzed the microbiome and serum metabolome of Yorkshire pigs to reveal the molecular regulation mechanism of average daily gain, which provided a valuable reference for the identification of ADG-related molecular markers in pig farming [20]. In cattle, the mechanisms of different daily weight gain were investigated by assessing their plasma amine/phenol- and carbonyl-metabolome and whole-blood immune gene expression profiles [12]. In addition, Ogunade et al. also used the plasma carboxyl-metabolome to analyze the mechanism of differences in the average daily gain of beef cattle [33]. However, it was reported that 90 percent of gut bacterial species were associated with 82 percent of fecal metabolites, while only 34 percent of bacterial species were associated with 24 percent of blood metabolites [34]. In contrast to serum-based omics methods, the fecal metabolome shows direct interactions among genetic, environmental, and dietary influences [35]. As a result, employing fecal samples in metabolomics research could enhance the identification of biomarkers. In the study, fecal metabolome analysis revealed significant alterations in the metabolites between the LADG and HADG. We identified 566 differentially abundant metabolites, including 123 upregulated and 443 downregulated metabolites, those enriched in purine metabolism, and secondary bile acid biosynthesis metabolic and other pathways. These metabolites may be more indicative of the metabolite profile of differences in average daily gain when combined with fecal microorganisms. Xu et al. analyzed the metabolites of average daily gain differences in the fecal metabolome of pre-weaning Holstein heifers and found 35 fecal metabolites with significant differences, of which 17 were up-regulated and 18 were down-regulated [36]. Nine metabolites were found to be significantly associated with ADG, including pretyrosine, 3-keto-b-D-galactose, 4-hydroxyglucobrassicin, venlafaxine, convalloside, D-fructose, (3R)-beta-leucine, nicoboxil, and dinoterb. The differentially abundant metabolites found in this study did not contain these nine metabolites, which may be related to the differences in breed and months of age of cattle.

Daily gain is a complex carcass trait, and a large part of the variation in average daily gain can be attributed to genetic factors, as average daily gain heritability in cattle has been reported to be moderate to high, between 0.30 and 0.6 [37]. The use of high-throughput RNA sequencing has been demonstrated to be a successful approach for gene expression studies [38]. Numerous transcriptomics studies related to productivity traits have been conducted in cattle, including skeletal muscle profiling among breeds [39], gene expression profiling among distinct muscle cuts [40], and heat stress response in blood [41]. In this research, we utilized RNA-seq technology to analyze the entire blood transcriptome of the LADG and HADG to identify their genetic distinctions. We identified 101 differentially expressed genes, including 45 upregulated and 56 downregulated genes. Enrichment analysis showed that the top 10 signaling pathways were alcoholism, systemic lupus erythematosus, viral carcinogenesis, collecting duct acid secretion, Rap1 signaling pathway, ECM–receptor interaction, PI3K-Akt signaling pathway, cell adhesion molecules, cGMP-PKG signaling pathway, and primary bile acid biosynthesis. One of the metabolic pathways is primary bile acid biosynthesis. Bile acids are cholesterol derivatives that play an important role in fat metabolism [42]. Primary bile acids are synthesized in the liver and converted to secondary and tertiary bile acids by intestinal bacterial flora [43]. Studies have shown that the ADG of production traits in livestock and poultry is related to bile acid metabolism, such as broilers [44,45]. These results suggest that the regulation of bile acid metabolism during beef cattle production may affect the ADG of cattle. *DPP6*, *CDKN1A*, and *FZD5* may be candidate genes for ADG variation in the transcriptomic analysis of high- and low-daily-gain Hanwoo cattle [6]. There was no difference in these genes in this study, which may be caused by different samples and varieties.

We performed joint analyses of the transcriptome, metabolome, and microbiome to systematically assess interdependencies at multiple levels, rather than looking at the omics individually, to identify the transcriptome–microbiome–metabolome relationships that are important for average daily gain. Differentially expressed genes were correlated with differentially abundant metabolites and indicator species. The genes that were significantly correlated with both differentially abundant metabolites and indicator species were *RADIL* and *ZBTB20*, and the microorganisms that were significantly correlated with *RADIL* and *ZBTB20* were *Oscillospira*. Fecal metabolomics is a functional reading of the gut microbiome that can reflect changes in the gut microbiome [9]. There are 20 metabolites related to *Oscillospira*. These metabolites were enriched and analyzed. The metabolic pathways involved included oxidative phosphorylation, arginine and proline metabolism, phenylalanine metabolism, and tyrosine metabolism. We then mapped the gene–microbial–metabolite network, the *RADIL*–*Oscillospira*–metabolite, by integrating transcriptomic, microbiome, and metabolomic data. *RADIL* is a downstream effector of Rap1, a universally expressed small GTPase that plays a key role in regulating cell adhesion, angiogenesis, and endothelial barrier function [46]. *Oscillospira* belongs to the family Ruminococcaceae, order Clostridiales, and class Clostridia in the phylum Firmicutes [47]. *Oscillospira* may be the next generation of candidate probiotics with great potential for health applications [48]. However, this study found that the abundance of *Oscillospira* at high daily gain was lower than that at low daily gain. Several studies have shown that there is a significant positive correlation between *Oscillospira* and low fat, leanness, and human health with weight loss, lipid reduction, and slimming [49]. This may explain that although *Oscillospira* is a probiotic, its abundance decreases at high daily growth. These results indicate that *RADIL* and *Oscillospira* could have potential application to estimate cattle with different ADG. But further larger-size studies with more various herds of cattle are desired to validate our finding. It is crucial to emphasize the variation in ADG from the collective influence of various factors working in unison. Although *RADIL* and *Oscillospira* were obtained by correlation analysis, it may be a key candidate gene and microbe, but not the only one. Host heredity is a key factor in determining the co-occurring microbiota in the host gut by host–microbial interaction [50]. This study revealed the possible potential role of genes–microbes–metabolites in high ADG cattle through Spearman correlation networks: *RADIL* activates bile acid metabolism by regulating *Oscillospira* and ultimately achieves higher daily gain, with precise mechanisms awaiting functional validation. This pattern of cross-level regulation explains the complexity that cannot be captured by single omics and provides a multi-dimensional intervention target for the targeted regulation of ADG.

This study also has limitations. Although our cohort (n = 30) exceeds typical single-omics studies, it remains insufficient to capture low-abundance microbes or rare metabolites, limiting generalizability. Furthermore, the exclusive focus on Angus steers precludes insights into breed or gender-specific ADG mechanisms. Future studies should include larger cohorts across breed, genders, and age groups to dissect genetic, developmental, and gender-specific mechanisms. Although Spearman networks revealed significant correlations, statistical associations do not imply causation and require further experimental verification.

## 4. Materials and Methods

### 4.1. Animals and Sample Collection

Fecal and serum samples were taken rectally from 30 Angus cattle from Inner Mongolia Muniuhai Livestock Development Co., Ltd. A total of 200 Angus cattle (all castrated males, aged 24.7 ± 1.44 months) were initially screened under standardized feeding conditions. Basal diet: 60% corn silage (DM basis), 30% concentrate (containing 16% crude protein, 5% fat, 2.5% calcium), 10% alfalfa hay. Cattle were ranked by ADG, and the top 7.5% (HADG, n = 15) and bottom 7.5% (LADG: n = 15) were selected to represent extreme phenotypes. The study involved cattle with an initial body weight (BW) of 210 ± 15 kg at weaning. The final BW was recorded as 550 ± 25 kg (HADG) and 480 ± 20 kg (LADG). All cattle were confirmed to be disease-free through veterinary inspection, and no antibiotics were administered during the trial. The study included only castrated male cattle to eliminate variations in growth related to gender. Following collection, fecal samples were collected in labeled containers, frozen in liquid nitrogen, and stored at −80 °C. Serum was isolated by centrifugation at 2000 rpm for 15 min at 4 °C and stored at −80 °C.

### 4.2. Amplicon Sequencing 16S rRNA and Subsequent Analysis

Microbial DNA was extracted by Guangzhou Magen Biotechnology, targeting the V3–V4 region of the 16S rRNA gene, with amplicons purified using AMPure XP Beads (Beckman Coulter, Brea, CA, USA). The 16S rRNA V3–V4-specific primers were 16S_F 5′-(CCTACGGGNGGCWGCAG)-3′ and 16S_R 5′-(GGACTACHVGGGTATCTAAT)-3′. Subsequently, the sequencing libraries were generated, and library quality was assessed. DNA library sequencing was performed on the Illumina NovaSeq 6000/PacBio Revivo platform by Gene Denovo Biotechnology Co., Ltd. (Guangzhou, China). Raw data containing adapters or low-quality reads would affect the following analysis. Thus, to obtain high-quality clean reads, raw reads were further filtered according to the following rules using FASTP (version 0.18.0): (1) removing reads containing more than 10% of unknown nucleotides (N), (2) removing reads containing more than 50% of bases with quality (Q-value) < 20, and (3) removing adapter contamination. The clean tags were clustered into operational taxonomic units (OTUs) of  ≥97% similarity using the UPARSE (version 9.2.64) pipeline. All chimeric tags were removed using the UCHIME algorithm and finally effective tags were obtained for further analysis. The tag sequence with the highest abundance was selected as a representative sequence within each cluster. The representative OTU sequences or ASV sequences were classified into organisms by a naive Bayesian model using the RDP classifier (version 2.2) based on the SILVA database (version 138.1) or the UNITE database (version 8.3), with a confidence threshold value of 0.8. The bioinformatics analysis was performed at www.omicshare.com.

### 4.3. Untargeted Metabolomics Study and Analysis

Fecal samples were subjected to untargeted metabolomics analyses. Fecal samples (100 mg) were dried overnight under a low-flow nitrogen stream to remove water. A total of 300 μL precooled acetonitrile and 100 μL precooled distilled water that acted as extraction solvent were added to each sample. Then, the mixture was vigorously vortexed for 3 min and sonicated for 5 min. After blending and centrifuging at 15,000 rpm for 15 min at 4 °C, the supernatant was obtained, and 10 μL of supernatant from each sample were mixed to obtain a quality control (QC) sample. Samples were filtered through a 0.22 μm microporous membrane for UHPLC-MS analysis. Analysis was performed using an UHPLC (1290 Infinity LC, Agilent Technologies, Waldbronn, Germany) coupled to a quadrupole time-of-flight (AB Sciex TripleTOF 6600, Shanghai, China). For HILIC separation, samples were analyzed using a 2.1 mm × 100 mm ACQUIY UPLC BEH Amide 1.7 µm column (waters, Wexford, Ireland). Solvent A was 0.1% formic acid in water and solvent B was 0.1% formic acid in acetonitrile. Metabolites were eluted using the following gradient at a flow of 0.4 mL/min: 0–2 min, 0–1% B; 2–6.5 min, 1–20% B; 6.5–11.5 min, 20–95% B; 11.5–13.5 min, 95–99% B; 13.5–16.5 min, 99–1% B; 16.5–20 min, 1% B; 20–21 min, 1–0% B; 21–22 min, 0% B. Injection volumes were set to 5 μL. Samples from all the groups were assayed in mixed and random order. Raw data underwent a conversion to the mzML format using Proteo Wizard software (version 3.0), followed by the extraction of peak data values using the XCMS program (version 3.18.0). The SIMCA-P program (version 14.1) was used to perform the analysis. A significant threshold of *p*-value ≤ 0.05 was set depending on the univariate T-test analysis. VIP > 1 and *p* < 0.05 were used to identify significantly changed metabolites between groups. The ultimate data in the current study were presented using www.omicshare.com.

### 4.4. RNA-Seq and Transcriptome Data Analysis

Total RNA was extracted from the blood samples following the instructions for TRlzol Reagent (Life Technologies, Carlsbad, CA, USA). The quality of RNA was assessed using a NanoDrop 2000 (Thermo Fisher Scientific, Wilmington, DE, USA), while the integrity of the RNA was evaluated using an Agilent Bioanalyzer 2100 system (Agilent Technologies, Santa Clara, CA, USA) with an RNA Nano 6000 detection kit. Biomarker Technologies Co., Ltd. (Beijing, China) carried out the construction of gene libraries and RNA-seq using qualified RNA samples. After the libraries passed quality control, we proceeded with PE150 pattern sequencing by utilizing the Illumina NovaSeq6000 sequencing platform. To obtain clean data, we employed perl scripting to remove reads that contained connectors and low-quality reads (reads with a proportion of N > 10% or a proportion of bases with Q ≤ 10 exceeding 50%) from the raw data. The clean data were compared with the reference genome (Bos_taurus_UMD_3.1.1) using Hisat2 version 2.0.4. Gene expression levels were quantified by fragments per kilobase per million reads (FPKM), and differential gene expression analysis of the two groups of samples was conducted through the utilization of DESeq2 version 1.30.1. Genes with a |log2 (fold change, FC)| > 1 and a false discovery rate (FDR) < 0.01 were deemed DEGs. The function of DEGs was annotated through databases such as Gene Ontology (GO) and the KEGG Ortholog database (KO). The DEGs underwent GO enrichment analysis and Kyoto Encyclopedia of Genes and Genomes (KEGG) pathway enrichment analysis using clusterProfiler version 4.4.4. Protein–protein interaction (PPI) detection was constructed for predicting present study-related multiple proteins and the visualizing based on the integrated data requesting from the STRING database (https://string-db.org/, v11.5, accessed on 1 December 2024).

### 4.5. Correlation Analysis of Microbiome, Metabolome, and Transcriptom

Using the Metware Cloud 1.0, a free online platform for data analysis (https://cloud.metware.cn, accessed on 12 December 2024), correlations between high- and low-daily-gain bovine differentially expressed genes and levels of various microorganisms and metabolites were analyzed and visualized. Correlation analysis was based on Spearman’s rank correlation coefficient |r|> 0.69 and *p* <0.05.

## 5. Conclusions

In summary, the microbiome, metabolome, and transcriptome of HADG and LADG Angus cattle were analyzed in this study. Different microorganisms, metabolites, and genes of the two groups were investigated to explore potential biomolecular markers affecting the average daily gain of Angus cattle. Compared with the LADG group, the abundance of Firmicutes increased in the HADG group, and the abundance of Bacteroidetes and Proteobacteria decreased. Metabolome and transcriptome analysis showed that the KEGG pathway involved in differentially expressed genes and differentially abundant metabolites was related to bile acid metabolism. Spearman’s correlation analysis showed that *Oscillospira* was positively correlated with *ZBTB20* and negatively correlated with *RADIL*. *ZBTB20* was negatively correlated with *dgA-11_gut_group*. This suggests a potential axis where Oscillospira modulates host gene expression to suppress fat deposition and enhance nutrient partitioning toward muscle growth, thereby improving ADG. The correlation network between gut microbiota (*Oscillospira*), host genes (*ZBTB20*, *RADIL*), and bile acid metabolites provides a foundation for developing machine learning models to predict ADG. In addition, *ZBTB20* and *RADIL* could serve as candidate genes for marker-assisted selection, accelerating genetic gains in beef cattle breeding programs. In summary, this multi-omics study reveals that the interplay between gut microbiota (*Oscillospira*), host genes (*ZBTB20*, *RADIL*), and bile acid metabolism underpins ADG divergence in Angus cattle. While these biomarkers show promise for precision breeding, their causal roles require further validation. The conclusions are derived from a single breed (Angus) and gender (castrated males). Future studies should include females and diverse breeds to assess generalizability.

## Data Availability

The original contributions presented in the study are included in the article; further inquiries can be directed to the corresponding author/s.
